# Comparison of the epidemiology of invasive pneumococcal disease between Australia and New Zealand in 2017–2021: an observational study based on surveillance data

**DOI:** 10.1016/j.lanwpc.2023.100764

**Published:** 2023-04-17

**Authors:** Nienke N. Hagedoorn, Andrew Anglemyer, Charlotte Gilkison, Mica Hartley, Tony Walls

**Affiliations:** a Department of Paediatrics, University of Otago, Christchurch, New Zealand; b Department of General Paediatrics, Erasmus MC Sophia Children's Hospital, Rotterdam, The Netherlands; c Health Intelligence Team, Institute of Environmental Science and Research, Wellington, New Zealand; d Department of Preventive and Social Medicine, University of Otago, Dunedin, New Zealand; e Communicable Diseases and Surveillance Section, Australian Government Department of Health, Canberra, Australia; f Research for Children Aotearoa—Wellbeing, Whanau, Health, Christchurch, New Zealand

**Keywords:** Invasive pneumococcal disease, Epidemiology, Vaccination, Pneumococcal conjugate vaccine, Serotype, Indigenous

## Abstract

**Background:**

The Australian immunisation schedule uses 13-valent pneumococcal conjugate vaccine (PCV13), while New Zealand (NZ) changed from PCV13 to 10-valent PCV (PCV10) in 2017. In NZ, cases of serotype 19A (not in PCV10) have been increasing since 2017. We compared invasive pneumococcal disease (IPD) epidemiology between Australia and NZ in 2017–2021.

**Methods:**

We collated IPD notification data from national surveillance systems. Between Australia and NZ, we compared IPD incidence rates and assessed the proportion of serotype 19A, and stratified for ethnicity and age.

**Findings:**

Between 2017 and 2021, the crude IPD incidence per 100,000 in Australia ranged from 4.3 to 8.4, and ranged from 6.9 to 11.4 in NZ. The highest age-adjusted IPD rates were observed in Australian Indigenous people (range: 27.3–35.5) followed by NZ Māori/Pacific peoples (range 19.7–30.4). For children <2 years, ethnicity-adjusted IPD rates were similar between Australia and NZ in 2017–2020. In 2021, however, the ethnicity-adjusted incidence in children <2 years was higher in NZ (30.2; 95% CI 21.1–39.4) than in Australia (23.3 95% CI: 19.5–27.1) (p < 0.01). In Australia, the proportion of serotype 19A remained 5%, whereas in NZ serotype 19A increased from 11.5% to 29.5% with the largest increase in children <2 years and 2–4 years.

**Interpretation:**

Despite higher risks in Indigenous populations in Australia compared to all other groups, the overall IPD rate in NZ is increasing, particularly among children. The numbers and proportions of IPD due to serotype 19A are increasing in NZ especially in children. These data support the NZ decision from December 2022 to change to PCV13.

**Funding:**

This research received no specific funding.

**Research in context**

**Evidence before this study**

We searched PubMed for articles on invasive pneumococcal disease using the search terms “*Streptococcus pneumoniae*”, “invasive pneumococcal disease”, “pneumococcal conjugate vaccine” and “impact”. We focused on studies published between January 1, 2002 and March 31, 2022 that studied the effect of 10-valent pneumococcal conjugate vaccine (PCV10) and 13-valent pneumococcal conjugate vaccine (PCV13) on invasive pneumococcal disease (IPD) incidence. In addition, we searched for studies based in the Asia–Pacific region.

Both PCV10 and PCV13 are known to reduce the incidence of IPD. Previous studies have not found any superiority for either vaccine in reducing the overall burden of pneumococcal disease. The choice of vaccine should be influenced by local epidemiology and serotype distribution. In a number of countries that use PCV10, infections due to serotype 19A have been increasing. A recent systematic review evaluating the burden of pneumococcal disease among adults found a large regional variation of IPD incidence and a higher burden in older populations. Prevalence of serotypes varied across Asia–Pacific countries, with serotype 3, serotype 19A, and serotype 19F as the most frequently reported serotypes. However, there are no studies comparing neighbouring countries, Australia and Aotearoa New Zealand (NZ) with regards to their IPD epidemiology. While similar in many ways, their different national immunisation programmes (NIPs) from 2017 to 2021, namely PCV13 in Australia and PCV10 in NZ, provide a unique opportunity to assess the potential impact of PCV10 and PCV13 NIPs, while taking into account their different population structures.

**Added value of this study**

In this observational study based on surveillance data of notifiable IPD, we found that incidence was higher in NZ compared to Australia 2017–2021, particularly in more recent years. The IPD incidence was highest in young infants and elderly. In addition, specific ethnic groups have a higher burden of IPD, including Australian Indigenous people and NZ Māori/Pacific peoples. In NZ, serotype 19A is the most abundant serotype and is increasing in all age groups and ethnic groups.

**Implications of all the available evidence**

These data support the decision to change the NIP in NZ starting from December 2022 to include PCV13. In NZ, additional pneumococcal immunisations for adult Māori/Pacific peoples or for elderly are unfunded. As the incidence of serotype 19A is quickly increasing in the elderly in NZ, surveillance efforts should continue to closely monitor this group to help inform future policy changes on pneumococcal vaccinations.

## Introduction

After introduction of the pneumococcal conjugate vaccine (PCV), invasive pneumococcal disease (IPD) and subsequent pneumococcal mortality has been reduced globally. Yet in 2015, pneumococcal infections are still estimated to cause 294,000 (range 192–366,000) deaths in HIV-uninfected children under 5 years.^1^ Some populations are at higher risk for IPD including young infants and specific ethnicities such as Australian Indigenous (Aboriginal and Torres strait islander), Māori or Pacific peoples.^2^^,^^3^

Several PCVs have been commercially available and cover different serotypes: 7-valent PCV (PCV7) (contains serotypes: 4, 6B, 9V, 14, 18C, 19F, and 23F) (Prevenar, Pfizer, no longer available), 10-valent PCV (PCV10) (contains PCV7 serotypes and serotypes: 1, 5, and 7F) (Synflorix, GSK), 13-valent PCV (PCV13) (contains PCV 10 serotypes and serotypes: 3, 6A, and 19A) (Prevenar 13, Pfizer), and 15-valent PCV (PCV15) (contains PCV13 serotypes and serotypes: 22F and 33F) (Vaxneuvance, Merck). Previous studies comparing the impact of PCV10 and PCV13 programmes until 2017 have not found superiority for either vaccine in the overall burden of pneumococcal disease.^4^ The World Health Organization recommends that a switch in the vaccine used in any national PCV programme should be considered if the epidemiology of IPD changes significantly.

**Fig. 1:**
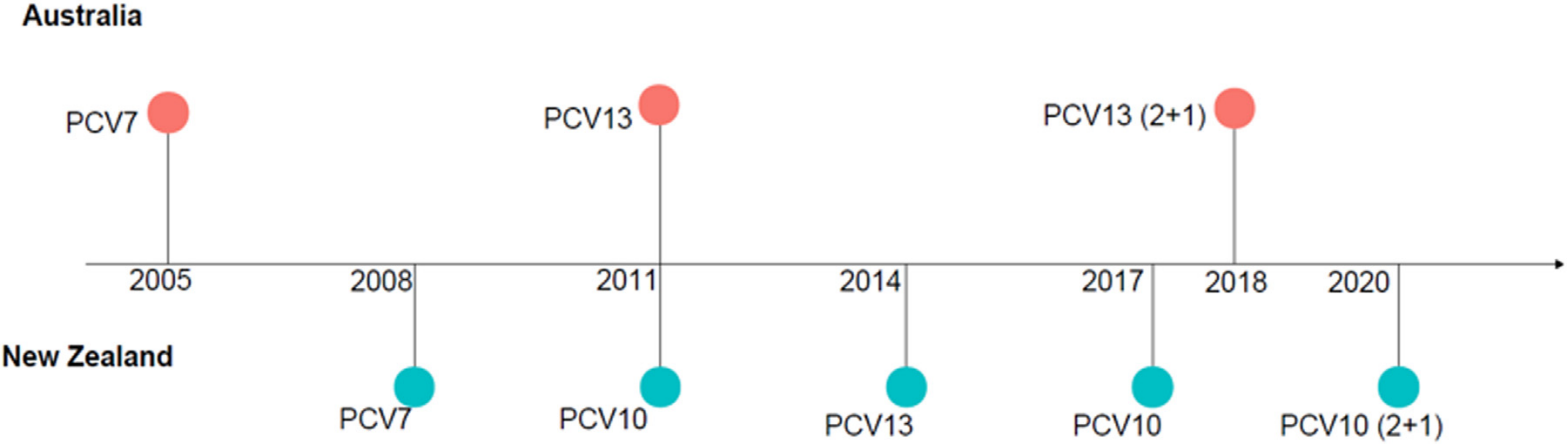
Important changes in universal immunisation schedules for children in Australia and New Zealand from 2005 to 2020.^5^^,^^6^ Australia: children with high-risk medical conditions and Indigenous children in high-incidence jurisdictions (Northern Territory, South Australia, Queensland, Western Australia): PCV13 in 3 primary doses + 1 booster schedule (3p + 1) and PPV23 at 4 years and 5 years. New Zealand: For children with high-risk medical conditions PCV13 in 3p + 1 schedule and 23PPV at 2 years and 7 years. PCV, pneumococcal conjugate vaccine; PPV, pneumococcal polysaccharide vaccine.

PCV13 has been used in the national immunisation programme (NIP) of Australia since 2011, initially with 3 primary doses, and then with 2 primary doses and 1 booster (2 + 1) since 2018. In Aotearoa New Zealand (NZ) PCV10 was used between 2011 and 2014, with a switch to PCV13 in 2014. In 2017 PCV10 was reintroduced, initially with a 3 + 1 schedule and then a 2 + 1 from July 2020 (Fig. 1).^5^^,^^6^ NZ children with high-risk medical conditions continued to be eligible for PCV13. Until 2019, full immunisation coverage at 12- and 24-months was >90% in both Australia and NZ, though historically full immunisation coverage at 12- and 24-months was lower for Australian Indigenous children (range 87.8%–92.9%) and NZ Māori (range 86.6%–91.6%).^7^^,^^8^ Since 2015, immunisation rates in NZ have been declining with increasing differences between ethnic groups.

When NZ switched to PCV10 in 2017 it was on the basis that immunogenicity studies suggested the inclusion of serotype 19F in PCV10 offered cross-protection for serotype 19A. In 2016, serotype 19A made up 17% of all IPD isolates in NZ.^9^ More recently, the evidence for any clinically significant impact of PCV10 cross-protection against serotype 19A has been questioned.^10^, ^11^, ^12^ A recent NZ study showed that the proportion of PCV10 preventable cases that are serotype 19A increased in all age groups from 45% in 2017 to 80% in 2020, with overall IPD incidence remaining similar.^11^ This trend of increasing serotype 19A infections has been noticed in several other countries around the world who use PCV10 on their childhood immunisation schedule.^13^, ^14^, ^15^, ^16^ Following the increase in serotype 19A isolates, Pharmaceutical Management Agency, PHARMAC (the NZ Crown entity governing which medicines and vaccines are subsidised) has decided to change to PCV13 in December 2022.^17^

A recent systematic review studied adult pneumococcal disease in the Asia–Pacific region, though NZ data in that study were limited and no country comparison was performed.^18^ To our knowledge, no previous study has compared IPD epidemiology in Australia and NZ. With similar experiences with regards to public health measures in response to the COVID-19 pandemic (e.g., pursuit of an elimination strategy in 2020 and most of 2021), Australia and neighbouring NZ present a unique opportunity to compare national level IPD epidemiology within two similar countries. Moreover, comparing IPD epidemiology in NZ and Australia, especially during the COVID-19 pandemic, is further supported as each country had similar strict public health measures, which globally have been associated with a reduction of bacterial infections such as IPD.^19^

In the present study, we aimed to compare the impact of two different NIPs in countries with similar healthcare systems and COVID-19 experiences, while considering their disparate population structures and risks. Namely, we assessed the IPD incidence within Australia (PCV13) and NZ (PCV10) in 2017–2021, with a focus on age, ethnic groups, and the role serotype 19A has played in the IPD incidence.

## Methods

### Study design and data collection

This is an observational study based on surveillance data from Australia and NZ between 1 January 2017 and 31 December 2021. We extracted IPD case notification data from national surveillance institutes (National Notifiable Diseases Surveillance System in Australia, and EpiSurv the national notifiable disease surveillance system operated by the Institute of Environmental Science and Research (ESR) in NZ) and merged those with census data derived from Australian Bureau of Statistics and Statistics New Zealand. In NZ, all diagnostic laboratories contribute data to the surveillance system and serotyping is performed at ESR's Invasive Pathogens Laboratory. In Australia, all laboratories contribute to the surveillance system and serotyping is performed by one of 4 reference laboratories, depending on which state the sample was collected including NSW Health pathology, PathWest Laboratory Medicine, Microbiological Diagnostic Unit Public Health Laboratory at the University of Melbourne, and Queensland Health. The Australian reference laboratories report their data to the National Notifiable Diseases Surveillance System.

IPD was defined as a laboratory confirmed infection of *S. pneumoniae* if isolated from a normally sterile site, detected with nucleic acid testing from a sterile site, or (in NZ only) a newer generation *S. pneumoniae* antigen test on cerebrospinal fluid or pleural fluid.

Age was grouped into <2 years, 2–4 years, 5–64 years and ≥65 years. Ethnicity was defined as Australian Indigenous (Aboriginal and Torres strait islander), Australian non-Indigenous, NZ Māori/Pacific peoples and NZ other ethnicities (which include NZ European, Asian, among others). Cases with missing ethnicity were excluded for this analysis (Australia: 7%, NZ: 1.3%).

### Data analysis

We estimated the crude annual incidence of IPD, which was expressed as incidence rates (IRs) per 100,000 and their 95% confidence intervals (CIs).^20^ We also estimated the crude IPD incidence stratified by age and ethnicity group within NZ and Australia to determine the country-specific burden of disease. To control for the effect different age distributions within each country could have on IPD risk, we estimated age-adjusted rates with direct standardisation, using the total study populations (Australia and NZ combined) as the standard population. To control for the effect different ethnic groups within each country could have on IPD risk, we estimated ethnicity-adjusted rates, with direct standardisation using the total study populations within each country as that country's standard population. The age- and ethnicity-adjusted IRs were compared by incidence rate ratios (IRR). We approximated the standard error of the age- and ethnicity-adjusted rates.^21^ Using the IRR, we tested whether the age- and ethnicity-adjusted rates were equal between Australia and New Zealand.^20^ Furthermore, to determine the influence of serotype 19A has had on IPD incidence within each country, we calculated the proportions of PCV10-specific serotypes and serotype 19A among all IPD cases, and stratified this by age and ethnicity. All analyses were performed in R version 4.2.2 (packages epiR, fmsb, ggplot2, tidyr). This research was approved by the ethics committee of University of Otago (HD22/029). In Australia, research that is based on non-identifiable data such as this project is deemed as negligible risk research and is therefore exempt of ethical review.^22^

### Role of the funding source

This research received no specific funding.

## Results

### Overall crude IPD rates

Between 2017 and 2021, the crude IPD rate in Australia ranged from 4.3 (95% CI 4.1–4.6) per 100,000 in 2020 to 8.4 (95% CI 8.0–8.8) per 100,000 in 2019 (Appendix 1: Table S1). In NZ, the crude IPD rate ranged from 6.9 (95% CI 6.2–7.6) per 100,000 in 2020 to 11.4 (95% CI 10.5–12.4) per 100,000 in 2018.

### Crude IPD rates by age and ethnicity

To determine the burden of disease by select risk groups within each country, we stratified crude IPD rates by both age and ethnicity (Appendix 2: Tables S2 and S3, Fig. S1). IPD incidence in NZ children <2 years increased from 2019 to 2021 in both NZ Māori/Pacific peoples (39.5 (95% CI 23.4–62.5) cases per 100,000 and 55.5 (95% CI 35.9–82.0) per 100,000 in 2019 and 2021, respectively) and NZ other (10.5 (95% CI 4.5–20.7) per 100,000 and 22.6 (95% CI 13.1–36.1) per 100,000 in 2019 and 2021, respectively) (Table S2 and Fig. S1). In contrast, the IPD incidence among Australian children <2 years remained similar in both Australian Indigenous people (51.4 (95% CI 31.4–79.3) per 100,000 and 44.3 (95% CI 26.2–70.0) per 100,000 in 2019 and 2021, respectively) and Australian non-Indigenous people (23.2 (95% CI 19.4–27.5) per 100,000 and 22.5 (95% CI 18.8–26.9) per 100,000 in 2019 and 2021, respectively) (Table S2 and Fig. S1).

Within Australia, IPD incidence between 2017 and 2021 in Australian Indigenous people was higher than in Australian non-Indigenous people in children <2 years (crude IRRs ranged 1.9–2.4), 5–64 years (crude IRRs ranged 7.3–18.7) and people ≥65 years of age (crude IRRs ranged 2.7–7.2) (Table S3). In NZ, we compared Māori/Pacific peoples to NZ other ethnicities and found that the incidence rates were consistently higher among Māori/Pacific peoples across age groups. IPD incidence among Māori/Pacific peoples compared to other ethnicities was higher in people 5–64 years (range IRRs 3.2–4.5), adults ≥65 years of age (range IRRs 3.1–5.5), and in children <2 years in 2019–2021 (range IRRs 2.5–3.8) (Table S3).

### Age- and ethnicity-adjusted IPD rates

**Fig. 2:**
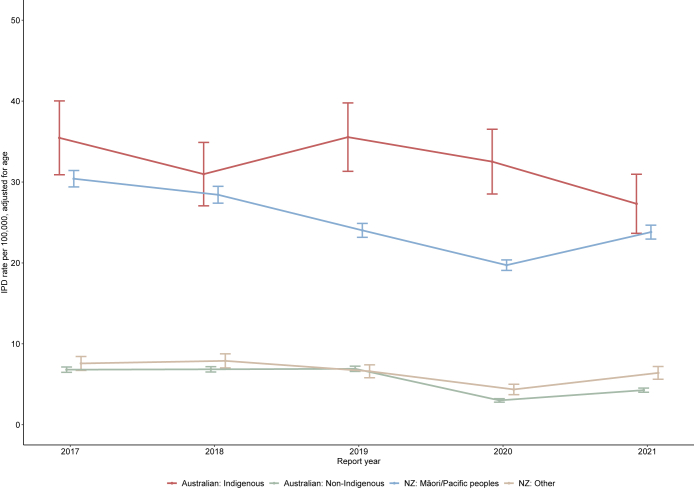
Age-adjusted IPD incidence rates and 95% CI by ethnicity groups and country, 2017–2021. Error bars indicate the 95% confidence interval. Incidence rates and incidence rate ratios are provided in Appendix 3, Tables S4 and S5. IPD, invasive pneumococcal disease; NZ, New Zealand.

The highest age-adjusted IRs were observed in Australian Indigenous people (ranging from 27.3 (95% CI 23.7–31.0) per 100,000 (in 2021) to 35.5 (95% CI 30.9–40.0) per 100,000 (in 2017)), followed by NZ Māori/Pacific peoples (ranging from 19.7 (95% CI 19.1–20.4) per 100,000 (in 2020) to 30.4 (95% CI 29.4–31.4) per 100,000 (in 2017)), NZ other (ranging from 4.4 (95% CI 3.7–5.0) per 100,000 (in 2020) to 7.9 (95% CI 7.0–8.8) per 100,000 (in 2018)) and Australian non-Indigenous people (ranging from 3.0 (95% CI 2.8–3.2) per 100,000 (in 2020) to 6.9 (95% CI 6.5–7.2) per 100,000 (in 2018/2019)) (Fig. 2, Appendix 3: Tables S4 and S5). The IRRs (95% CI) comparing the age-adjusted IRs between Australian Indigenous people and Australian non-Indigenous people ranged from 4.5 (4.3–4.7) per 100,000 in 2018 to 10.8 (10.1–11.6) per 100,000 in 2020 (Table S5). The IRRs (95% CI) comparing NZ Māori/Pacific peoples and NZ other ranged from 3.6 (3.4–3.8) per 100,000 in 2018/2019 to 4.5 (4.3–4.8) per 100,000 in 2020 (Table S5).

**Fig. 3:**
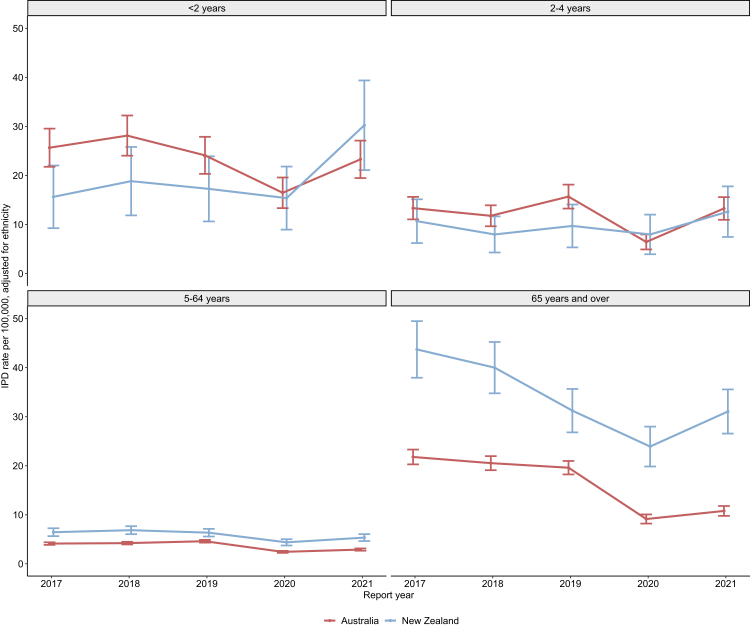
Ethnicity-adjusted incidence rate of IPD per 100,000 by age group and country, 2017–2021. Error bars indicate the 95% confidence interval. Incidence rates and incidence rate ratios are provided in Appendix 4, Tables S6 and S7. IPD, invasive pneumococcal disease; NZ, New Zealand.

To control for the effects of different risks as they pertain to disparate ethnic groups, we also estimated ethnicity-adjusted rates within each country (Fig. 3, Appendix 4: Tables S6 and S7). Generally, across the different age groups, ethnicity-adjusted IPD incidence rates were highest in those <5 years and those ≥65 years. Further, the ethnicity-adjusted incidence rates were higher in NZ than in Australia in people ≥65 years and people from 5 to 64 years (p-value < 0.001) (Fig. 3, Tables S6 and S7). Ethnicity-adjusted incidence rates among children aged 2–4 years of age did not differ between Australia and NZ. For children <2 years of age, ethnicity-adjusted IPD incidence rates were higher in Australia compared to NZ in 2017 and 2018, and were similar in 2019–2020. In 2021, however, the ethnicity-adjusted incidence of IPD in children <2 years was higher in NZ (adjusted IR: 30.2 (95% CI 21.1–39.4)) than in Australia (adjusted IR: 23.3 (95% CI: 19.5–27.1)) (IRR 1.3 (95% CI 1.2–1.4)) (p < 0.01) (Fig. 3, Tables S6 and S7).

### PCV10 serotypes and serotype 19A

**Fig. 4:**
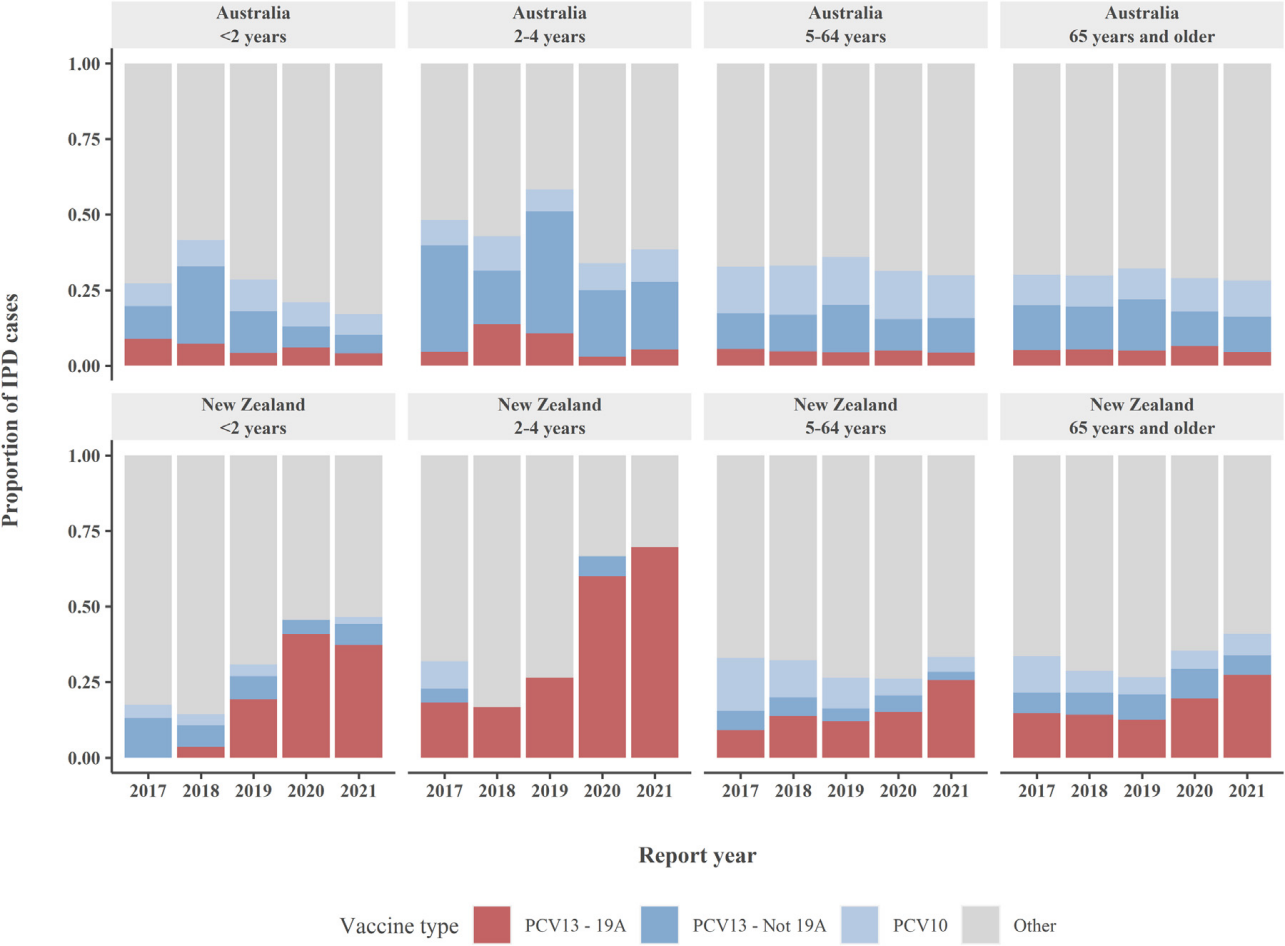
Percentage of annual IPD cases by vaccine type, age group and country, 2017–2021. Numbers and proportion of serotype 19A shown in Appendix 3. IPD, invasive pneumococcal disease; PCV, pneumococcal conjugate vaccine.

In Australia, the proportion of PCV10 serotypes and of serotype 19A were 12% and 5%, respectively, and the proportions did not vary over the years (Fig. 4; Appendix 5: Tables S8–S10; Appendix 6: Fig. S2). A similar trend was observed across the different age groups. In Australian Indigenous people, a higher proportion of PCV10 serotypes (range 15.8–20.6%) was observed compared to Australian non-Indigenous people (range 10.8–12.3%), whereas the proportion of 19A was lower in Australian Indigenous people (range 0.8–3.2% vs 4.9–6.3%).

In NZ, during the study period the proportion of PCV10 serotypes decreased from 14.2% (74/521) to 5.3% (25/468) and the proportion of serotype 19A increased from 11.5% (60/521) to 29.5% (138/468) in 2021 (Table S8). This increase in proportion of serotype 19A occurred in all ethnicity and age groups (Tables S9 and S10). The largest increase of serotype 19A was observed in children <2 years (0.0% (0/23)–37.2% (16/43)) and 2–4 years (18.2% (4/22)–69.6% (16/23)) (Table S9). In 2021, the proportion of disease due to serotype 19A of all serotypes included in PCV13 and PCV10 was 100% (16/16) in children 2–4 years of age, and 76% (16/20) in children <2 years of age.

### Preliminary results for 2022

Early assessments of preliminary data for 2022 (up to July) suggest a widening gap between NZ and Australia, particularly among children <2 years of age. The crude rate of IPD in NZ children <2 years of age was more than three-times higher than in Australia (36.8 per 100,000 in the 12 months ending June, with 64.3% of isolates serotype 19A). In Australia the crude rate of IPD in children <2 years of age was 11.3 per 100,000 with 1.5% of isolates serotype 19A.

## Discussion

In this comparison study of IPD epidemiology in Australia and NZ, we found that the crude incidence of IPD was higher in NZ compared to Australia during the years 2017–2021. Our data confirm that Australian Indigenous people are disproportionally affected by IPD, followed by NZ Māori/Pacific peoples. Despite higher risks in Indigenous populations in Australia compared to all other groups, the overall IPD rate in NZ is increasing, particularly among children. In NZ, the high increase of serotype 19A occurred in all ethnic groups and age groups whereas in Australia the proportion of serotype 19A remained low and stable over time.

Comparing the IPD incidence to other high-income countries, the IPD incidence in Australia and NZ was similar to the IPD incidence in America (2019: IR 9.2), but was higher compared to European countries (2018: average 6.4, range per country 0.5–13.9).^23^^,^^24^ We focused our analysis on the 2017–2021 time period as we were interested in comparing the PCV10 and PCV13 schedule between Australia and NZ. We acknowledge that NZ had higher IPD incidence (range IR 9.7–10.9) than Australia (range IR 6.3–8.3) in the timeframe 2014–2017 when Australia and NZ both used PCV13,^9^^,^^25^ though PCV10 was also used in NZ 2011–2014 and many children in that era would have been vaccinated under a previous NIP. Differences in IPD incidence could be influenced by differences in NIPs, surveillance systems, serotype epidemiology and population characteristics such as age distribution and proportion of indigenous populations. In New Zealand, for instance, ∼15% of people are of Māori ethnicity compared Australia where ∼3% of people are of Australian Indigenous ethnicity.

The emergence of serotypes not included in vaccines following introduction of PCVs is described globally and is called serotype replacement.^26^ Serotype replacement is a complex phenomenon which depends on regional factors, immunisation coverage, serotype invasiveness, and antibiotic pressure.^27^ Serotype 19A is currently the most abundant serotype in NZ and is increasing in all age groups. Similar to NZ, increased incidence of serotype 19A has been reported after introduction of PCV10 in Belgium, Brazil, Colombia, and Chile.^13^^,^^16^^,^^28^^,^^29^ On the other hand, other countries (e.g., The Netherlands and Finland) did not see any changes in serotype 19A incidence after the introduction of PCV10 while South Korea and Israel were seeing an increase before they introduced PCV7, which also does not offer protection against serotype 19A.^30^, ^31^, ^32^, ^33^

One large study showed that IPD incidence decreased globally during the COVID-19 pandemic in 2020.^19^ Similarly, our study showed a drop in IPD incidence in Australia (IR 8.4–4.3) and NZ (IR 9.9–6.9) from 2019 to 2020, though the rate of IPD due to serotype 19A continued to increase in 2020 in NZ.^11^ In Australia, these reductions in IPD following public health restrictions occurred predominantly in Australian non-Indigenous people whereas the IPD rate in Australian Indigenous people remained relatively stable. This might indicate the in Australian Indigenous people other risk factors are involved. In 2021, IPD incidence increased again in NZ (IR = 9.2), although in Australia IPD incidence remained relatively stable (IR = 5.2). Interestingly, in NZ the proportion of all isolates that were serotype 19A increased sharply from 2019 to 2021.

In July 2020, NZ switched from a schedule of three primary doses with no booster (3 + 0) to a schedule with two primary doses with 1 booster (2 + 1) for PCV10. It is yet unclear whether this schedule change will influence IPD incidence or 19A incidence. It is suggested though that the 2 + 1 schedule provides higher antibodies and may provide longer protection compared to the 3 + 0 schedule.^4^

Similar to other countries, a decline in immunisation coverage during the COVID-19 pandemic was observed in NZ.^34^ In 2020, childhood immunisation coverage declined in NZ (fully coverage at 12 months 80.4%) and increased again in 2021 (fully coverage at 12 months 90.4%).^8^ Concerningly, differences in immunisation coverage across ethnic groups increased with lowest coverage in NZ Māori (fully coverage at 12 months 66.2% in 2020 and 81.5% in 2021). In Australia, however, immunisation coverage did not decline in 2020 or 2021.^7^ In NZ, the impact of the rapid decrease in immunisation rates in 2020 has not yet been observed in IPD incidence among children <5 years. Of children <5 diagnosed with IPD in 2022, more than 80% were either fully vaccinated or on schedule for their age at time of diagnosis.^35^ Nevertheless, overall immunisation coverage should be improved including catch-up immunisations after the decrease in immunisation coverage in 2020.

Similar to other studies, our data confirm that Australian Indigenous people and NZ Māori/Pacific peoples are disproportionally affected by IPD.^2^^,^^3^ During 2017–2021 this gap has not been reduced. Australian Indigenous still have over >4 fold higher-risk compared to Australian non-Indigenous, while NZ Māori/Pacific peoples have a >2.5 fold higher-risk of acquiring IPD compared to NZ other ethnicities. This gap might be explained by a lower immunisation coverage, although individual level immunisation data was not available in Australia. Besides immunisation status, other risk factors for IPD should be taken into account including lower socio-economic status, or high medical risk comorbidities. In addition, health care accessibility should be ensured for all ethnic groups.

In our study, the largest difference in ethnicity was observed in people 5–64 years of age and ≥65 years of age. Although overall IPD incidence decreased in these age groups, the burden of disease in people ≥65 years of age was substantial (2021: Australia IR 10.2, NZ IR 23.1). In Australia, people >70 years are eligible for PCV13 and Indigenous people >50 years are eligible for PCV13 and a 23-valent pneumococcal polysaccharide vaccine (23-PPV). In contrast, NZ does not fund additional pneumococcal immunisations for adult Māori/Pacific peoples or for elderly.

As serotype 19A is increasing in the elderly, NZ surveillance should monitor serotype and IPD incidence in this group to inform policy on pneumococcal vaccinations. Further, the burden of disease should be considered as the influence of infant vaccinations can provide indirect immunity in older age groups.

Should countries switch to a higher-valent PCV and when? The statement by the World Health Organization recommends only switching when the epidemiology of IPD changes significantly.^4^ In NZ, the trend of increasing cases of serotype 19A together with the increase of IPD incidence in children <2 years of age is concerning. Several countries have not seen an increase in serotype 19A after PCV13 implementation in the immunisation programme.^10^

Starting in December 2022, PHARMAC in NZ changed the infant immunisation schedule to PCV13. It is likely that a significant decrease in IPD incidence and serotype 19A incidence following this change from PCV10 will take a few years to be observed. In particular, catch-up immunisation for children <5 years is not part of the PCV programme change, therefore those who received PCV10 are still insufficiently protected from serotype 19A. Lastly, improving immunisation coverage, particularly in high-risk groups, should be a key aspect of any plan to reduce the impact of IPD due to serotype 19A.

The main strength of our study is that surveillance data of IPD is of high-quality both in Australia and NZ. Also, we used a highly specific laboratory-confirmed definition of IPD. Although this definition does not represent the total disease burden of IPD, it ensures our results are not overestimated. This study has some limitations. First, the NZ case definition for IPD included detection of *S. pneumoniae* by newer generation antigen tests whereas the Australian case definition did not. As the number of cases detected by antigen tests was <2%, this has unlikely influenced our results. Second, we used aggregated data and individual data on medical history or immunisation status was not available, particularly in Australia. We acknowledge that children at high medical risk were previously eligible for PCV13 in NZ (of the 97 PCV-eligible children who were diagnosed with 19A in 2021 and 2022 in NZ, only 2 received PCV13 alone)^35^^,^^36^ and for 23- pneumococcal polysaccharide vaccine in Australia. Third, we acknowledge that Australian Indigenous children in some jurisdictions are eligible for 23-PPV. This impact was negligible as exclusion of this group in the analysis did not influence our results (not shown). Fourth, due to low numbers of IPD in Māori and Pacific peoples it was not possible to analyse those ethnic groups separately. Lastly, since only overall vaccine coverage, including all immunisations was available, it was not possible to analyse the impact of different pneumococcal vaccines on IPD incidence. Therefore, we focused our analysis on comparison of incidence rates and proportion of serotype 19A between Australia and NZ.

### Conclusion

In this study based on surveillance data of IPD in Australia and NZ between 2017 and 2021, Australian Indigenous people followed by NZ Māori/Pacific peoples have a disproportionally higher burden of IPD. Despite higher risks in Indigenous populations in Australia compared to all other groups, the overall IPD rate in NZ is increasing, particularly among children. In addition, in NZ serotype 19A is the most common serotype and is increasing in all age and ethnic groups. Especially in NZ children <2 years, IPD incidence increased sharply, explained largely by the increase in serotype 19A. Our study supports the decision to change from PCV10 to PCV13 in NZ.

## Contributors

Conceptualisation: N.N.H., T.W., A.A.

Methodology: N.N.H., A.A., C.G., M.H., T.W.

Formal analysis: N.N.H., A.A., C.G.

Data curation: A.A., M.H.

Writing – Original draft: NH, Writing – Review & Editing: A.A., C.G., M.H., T.W.

## Data sharing statement

The aggregate data underlying the study is presented in the Supplementary material. Queries about data availability may be directed to: survqueries@esr.cri.nz.

## Ethics committee approval

This research was approved by the ethics committee of University of Otago (HD22/029). In Australia, research that is based on non-identifiable data such as this project is deemed as negligible risk research and is therefore exempt of ethical review.

## Declaration of interests

There are no declared conflicts of interest.

## Appendix A Supplementary data

Appendix Tables S1–S10 and Figs. S1 and S2

IPD Protocol
