# Supplementary material for: Comparison of the epidemiology of invasive pneumococcal disease between Australia and New Zealand in 2017–2021: an observational study based on surveillance data
Source: Lancet Reg Health West Pac. 2023 Apr 17;36:100764. doi: 10.1016/j.lanwpc.2023.100764 (PMC10398586; doi:10.1016/j.lanwpc.2023.100764)

Supplementary material

Appendix 1 – Annual crude IPD incidence rates 2017-2021, Australia and New Zealand

Table S1: IPD incidence rate (per 100,000)

Table S1: IPD incidence rate (per 100,000)

	Australia	New Zealand
	IR (95% CI)	IR (95% CI)
2017	8·3 (8-8·7)	10·9 (10-11·9)
2018	8·1 (7·8-8·5)	11·4 (10·5-12·4)
2019	8·4 (8-8·8)	9·9 (9·1-10·9)
2020	4·3 (4·1-4·6)	6·9 (6·2-7·6)
2021	5·2 (4·9-5·5)	9·2 (8·4-10·1)

CI, confidence interval; IPD, invasive pneumococcal disease; IR, incidence rate

Appendix 2 – Annual crude IPD incidence rates and incidence rate ratios stratified for age and ethnicity, 2017-2021, Australia and New Zealand

Table S2: IPD incidence rate stratified for ethnicity and age

	<2 years	2-4 years	5-64 years	65 years and over
**Australian Indigenous**				
	IR (95% CI)	IR (95% CI)	IR (95% CI)	IR (95% CI)
2017	47·7 (28·3-75·5)	17·8 (8·5-32·7)	25·0 (21·4-29·1)	91·1 (62·7-128)
2018	65·6 (42·5-96·9)	23·1 (12·3-39·6)	26·1 (22·5-30·2)	51·7 (31·6-79·8)
2019	51·4 (31·4-79·3)	21·2 (11·0-37·1)	30·2 (26·3-34·5)	63·0 (41·1-92·3)
2020	30·2 (15·6-52·7)	21·1 (10·9-36·8)	28·6 (24·8-32·7)	54·5 (34·9-81·1)
2021	44·3 (26·2-70·0)	17·3 (8·3-31·9)	22·2 (18·9-25·9)	50·8 (32·6-75·7)
**Australian non-Indigenous**				
	<2 years	2-4 years	5-64 years	65 years and over
2017	24·9 (21-29·3)	13·2 (10·9-15·8)	3·4 (3·2-3·7)	19·4 (18-20·9)
2018	26·8 (22·8-31·4)	11·4 (9·3-13·8)	3·5 (3·2-3·7)	19·5 (18·1-20·9)
2019	23·2 (19·4-27·5)	15·5 (13·0-18·3)	3·7 (3·5-4·0)	18·1 (16·8-19·5)
2020	16·0 (12·9-19·7)	5·9 (4·4-7·8)	1·5 (1·4-1·7)	7·6 (6·7-8·4)
2021	22·5 (18·8-26·9)	13·1 (10·8-15·8)	2·2 (2-2·4)	9·4 (8·5-10·3)
**NZ Māori/pacific**				
	<2 years	2-4 years	5-64 years	65 years and over
2017	24·4 (12·6-42·6)	10·6 (4·6-20·8)	13·8 (11·5-16·5)	121 (95-151·9)
2018	30·5 (17·0-50·2)	11·9 (5·4-22·5)	15·3 (12·8-18·1)	98 (75·1-125·6)
2019	39·5 (23·4-62·5)	13·1 (6·0-24·9)	15·7 (13·3-18·4)	65·4 (48·2-86·7)
2020	31·1 (17·0-52·2)	8·7 (3·2-18·9)	10·7 (8·7-12·9)	64·0 (47·3-84·6)
2021	55·5 (35·9-82·0)	11·6 (5·0-22·8)	12·3 (10·2-14·7)	75·7 (57·5-97·9)
**NZ Other**				
	<2 years	2-4 years	5-64 years	65 years and over
2017	13·2 (6·6-23·6)	10·7 (5·9-18)	4·4 (3·7-5·2)	22·1 (18·7-26)
2018	15·6 (8·3-26·7)	6·9 (3·2-13·1)	4·5 (3·8-5·3)	23·9 (20·3-27·8)
2019	10·5 (4·5-20·7)	8·7 (4·2-16)	3·5 (2·9-4·3)	20·8 (17·6-24·5)
2020	10·6 (4·6-20·9)	7·8 (3·5-14·7)	2·5 (1·9-3·1)	11·8 (9·4-14·6)
2021	22·6 (13·1-36·1)	12·9 (7·2-21·3)	3·2 (2·6-4·0)	17·5 (14·6-20·8)

CI, confidence interval; IPD, invasive pneumococcal disease; IR, incidence rate; NZ, New Zealand

Table S3: Crude IPD incidence rate ratios stratified for ethnicity and age

	<2 years	2-4 years	5-64 years	65 years and over
	**Australian Indigenous vs Australian non-Indigenous**			
	IRRs (95% CI)	IRRs (95% CI)	IRRs (95% CI)	IRRs (95% CI)
2017	1·9 (1·2-3·1)	1·3 (0·7-2·6)	7·3 (6·2-8·7)	4·7 (3·3-6·7)
2018	2·4 (1·6-3·7)	2·0 (1·1-3·6)	7·5 (6·4-8·9)	2·7 (1·7-4·1)
2019	2·2 (1·4-3·5)	1·4 (0·8-2·5)	8·1 (7·0-9·4)	3·5 (2·4-5·1)
2020	1·9 (1·0-3·4)	3·6 (1·9-6·6)	18·7 (15·6-22·3)	7·2 (4·8-10·9)
2021	2·0 (1·2-3·2)	1·3 (0·7-2·5)	10·0 (8·3-12)	5·4 (3·6-8·2)
	**NZ Māori/pacific vs NZ other**			
	<2 years	2-4 years	5-64 years	65 years and over
	IRRs (95% CI)	IRRs (95% CI)	IRRs (95% CI)	IRRs (95% CI)
2017	1·8 (0·8-4·2)	1·0 (0·4-2·3)	3·2 (2·5-4·0)	5·5 (4·1-7·2)
2018	2·0 (0·9-4·1)	1·7 (0·7-4·3)	3·4 (2·7-4·3)	4·1 (3·1-5·5)
2019	3·8 (1·6-8·7)	1·5 (0·6-3·7)	4·5 (3·5-5·7)	3·1 (2·3-4·4)
2020	2·9 (1·2-7·0)	1·1 (0·4-3·1)	4·3 (3·2-5·8)	5·4 (3·8-7·7)
2021	2·5 (1·3-4·6)	0·9 (0·4-2·1)	3·8 (2·9-5)	4·3 (3·2-5·9)

CI, confidence interval; IPD, invasive pneumococcal disease; IRR, incidence rate ratios; NZ, New Zealand

Figure S1. Annual crude IPD incidence by ethnic group and age group, 2017–2021

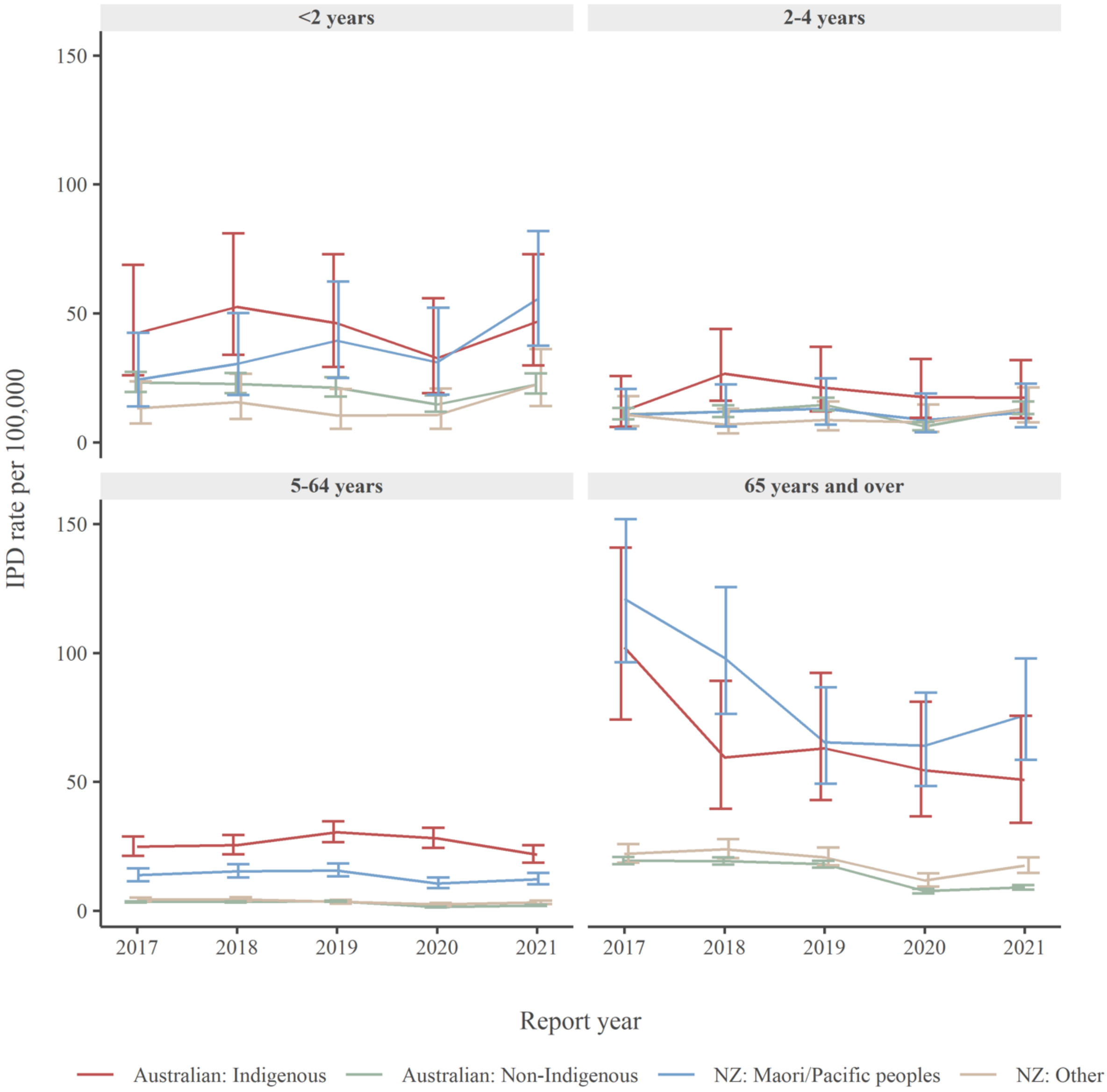

Appendix 3 – Annual IPD incidence rates and incidence rate ratios standardised for age, stratified for ethnicity, 2017-2021, Australia and New Zealand

Table S4: IPD incidence rate (per 100,000) for ethnicity, standardised for age

	Australian indigenous	Australian non-indigenous	NZ Māori/Pacific	NZ Other
	IR (95% CI)	IR (95% CI)	IR (95% CI)	IR (95% CI)
2017	35·5 (30·9-40·0)	6·8 (6·5-7·1)	30·4 (29·4-31·4)	7·6 (6·7-8·4)
2018	31·0 (27·1-34·9)	6·9 (6·5-7·2)	28·4 (27·4-29·5)	7·9 (7·0-8·8)
2019	35·5 (31·3-39·8)	6·9 (6·6-7·2)	24·0 (23·2-24·9)	6·6 (5·8-7·4)
2020	32·5 (28·5-36·5)	3·0 (2·8-3·2)	19·7 (19·1-20·4)	4·4 (3·7-5·0)
2021	27·3 (23·7-31·0)	4·3 (4·0-4·5)	23·8 (22·9-24·7)	6·4 (5·6-7·2)

CI, confidence interval; IPD, invasive pneumococcal disease; IR, incidence rate; NZ, New Zealand

Table S5: IPD incidence rate ratios for ethnicity, based on standardised rates for age

	Australian indigenous vs Australian non-Indigenous	NZ Māori/Pacific peoples vs NZ Other
	IRR (95%CI)	IRR (95%CI)
2017	5·2 (5·0-5·5)	4·0 (3·8-4·2)
2018	4·5 (4·3-4·7)	3·6 (3·4-3·8)
2019	5·1 (4·9-5·4)	3·6 (3·5-3·8)
2020	10·8 (10·1-11·6)	4·5 (4·3-4·8)
2021	6·4 (6·0-6·8)	3·7 (3·5-3·9)

CI, confidence interval; IRR, incidence rate ratios; NZ, New Zealand

Appendix 4 – Annual IPD incidence rates and incidence rate ratios standardised for ethnicity, stratified for age, 2017-2021, Australia and New Zealand

Table S6: IPD incidence rate (per 100,000) standardised for ethnicity, stratified for age

**Australia**				
	<2 years	2-4 years	5-64 years	65 years and over
	IR (95% CI)	IR (95% CI)	IR (95% CI)	IR (95% CI)
2017	25·7 (21·8-29·6)	13·3 (11-15·6)	4·1 (3·9-4·4)	21·8 (20·3-23·3)
2018	28·1 (24-32·2)	11·8 (9·6-13·9)	4·2 (4·0-4·5)	20·5 (19·1-22·0)
2019	24·1 (20·3-27·9)	15·7 (13·3-18·1)	4·6 (4·3-4·9)	19·6 (18·2-21·0)
2020	16·5 (13·3-19·6)	6·4 (4·9-8·0)	2·4 (2·2-2·6)	9·1 (8·2-10·1)
2021	23·3 (19·5-27·1)	13·3 (11·0-15·6)	2·9 (2·7-3·1)	10·8 (9·8-11·8)
**New Zealand**				
	<2 years	2-4 years	5-64 years	65 years and over
	IR (95% CI)	IR (95% CI)	IR (95% CI)	IR (95% CI)
2017	15·6 (9·3-22)	10·7 (6·2-15·1)	6·4 (5·6-7·3)	43·7 (37·9-49·5)
2018	18·8 (11·9-25·8)	8·0 (4·3-11·7)	6·9 (6·0-7·7)	40·0 (34·8-45·2)
2019	17·3 (10·6-23·9)	9·7 (5·3-14·1)	6·4 (5·6-7·1)	31·2 (26·8-35·7)
2020	15·4 (9·0-21·8)	8·0 (3·9-12·0)	4·4 (3·7-5·0)	23·9 (19.9-28·0)
2021	30·2 (21·1-39·4)	12·6 (7·5-17·8)	5·3 (4·6-6·1)	31·1 (26·6-35·6)

CI, confidence interval; IPD, invasive pneumococcal disease; IR, incidence rate; y, years

Table S7: IRRs based on ethnicity-adjusted rates, stratified for age; New Zealand vs Australia

	<2 years	2-4 years	5-64 years	65 years and over
	IRR (95% CI)	IRR (95% CI)	IRR (95% CI)	IRR (95% CI)
2017	0·6 (0·6-0·7)	0·8 (0·7-0·9)	1·6 (1·4-1·8)	2·0 (1·9-2·1)
2018	0·7 (0·6-0·7)	0·7 (0·6-0·8)	1·6 (1·4-1·8)	1·9 (1·8-2·1)
2019	0·7 (0·7-0·8)	0·6 (0·6-0·7)	1·4 (1·2-1·6)	1·6 (1·5-1·7)
2020	0·9 (0·9-1·0)	1·2 (1·1-1·4)	1·8 (1·5-2·1)	2·6 (2·4-2·8)
2021	1·3 (1·2-1·4)	1·0 (0·9-1·0)	1·8 (1·6-2·1)	2·9 (2·7-3·1)

IRR, incidence rate ratio; CI, confidence interval

Appendix 5 – Proportions of serotype 19A for all IPD cases, overall, stratified for age, stratified for ethnicity, 2017-2021, Australia and New Zealand

Table S8: Proportion of serotype 19A

	**Australia**	**New Zealand**
	serotype 19a/IPD cases (%)	serotype 19a/IPD cases (%)
2017	114/2044 (5·6%)	60/521 (11·5%)
2018	116/2030 (5·7%)	75/557 (13·5%)
2019	105/2131 (4·9%)	65/495 (13·1%)
2020	60/1112 (5·4%)	71/350 (20·3%)
2021	61/1339 (4·6%)	138/468 (29·5%)

IPD, invasive pneumococcal disease

Table S9: Proportion of serotype 19A stratified for age

	**Australia**			
	<2 years	2-4 years	5-64 years	65 years and over
	serotype 19a/IPD cases (%)	serotype 19a/IPD cases (%)	serotype 19a/IPD cases (%)	serotype 19a/IPD cases (%)
2017	14/167 (8·4%)	8/130 (6·2%)	50/953 (5·2%)	42/794 (5·3%)
2018	14/181 (7·7%)	15/117 (12·8%)	44/941 (4·7%)	43/791 (5·4%)
2019	6/156 (3·8%)	19/159 (11·9%)	43/1037 (4·1%)	37/779 (4·7%)
2020	6/107 (5·6%)	2/68 (2·9%)	28/568 (4·9%)	24/369 (6·5%)
2021	6/143 (4·2%)	7/126 (5·6%)	27/629 (4·3%)	21/441 (4·8%)
	**New Zealand**			
	<2 years	2-4 years	5-64 years	65 years and over
2017	-	4/22 (18·2%)	23/252 (9·1%)	33/224 (14·7%)
2018	1/28 (3·6%)	3/18 (16·7%)	38/277 (13·7%)	33/234 (14·1%)
2019	5/26 (19·2%)	5/19 (26·3%)	31/258 (12·0%)	24/192 (12·5%)
2020	9/22 (40·9%)	9/15 (60·0%)	27/180 (15·0%)	26/133 (19·5%)
2021	16/43 (37·2%)	16/23 (69·6%)	56/219 (25·6%)	50/183 (27·3%)

IPD, invasive pneumococcal disease

Table S10: Proportion of serotype 19A stratified for ethnicity

	Australian Indigenous	Australian non-Indigenous	NZ Māori/Pacific	NZ Other
	serotype 19a/IPD cases (%)	serotype 19a/IPD cases (%)	serotype 19a/IPD cases (%)	serotype 19a/IPD cases (%)
2017	3/232 (1·3%)	102/1627 (6·3%)	16/214 (7·5%)	43/298 (14·4%)
2018	2/240 (0·8%)	105/1663 (6·3%)	27/221 (12·2%)	46/318 (14·5%)
2019	3/272 (1·1%)	95/1706 (5·6%)	24/228 (10·5%)	40/264 (15·2%)
2020	8/254 (3·1%)	45/751 (6·0%)	33/175 (18·9%)	38/175 (21·7%)
2021	6/215 (2·8%)	52/1066 (4·9%)	55/213 (25·8%)	83/254 (32·7%)

IPD, invasive pneumococcal disease

Appendix 6 – Proportion of annual IPD cases by vaccine type and country, 2017-2021, Australia and New Zealand

Figure S2. Proportion of annual IPD cases by vaccine type and country, 2017-2021, Australia and New Zealand

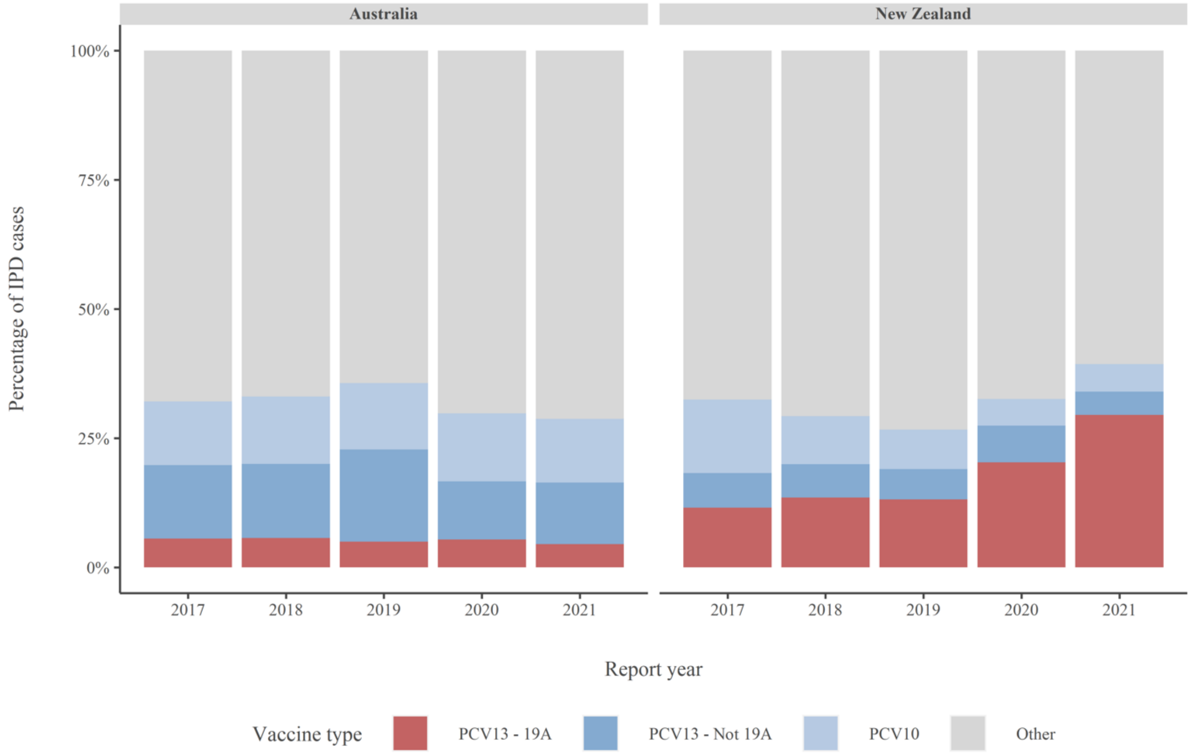

Supplement: Appendix Tables S1–S10 and Figs. S1 and S2 [file mmc1.pdf]
