# Supplementary material for: Comparison of the epidemiology of invasive pneumococcal disease between Australia and New Zealand in 2017–2021: an observational study based on surveillance data
Source: Lancet Reg Health West Pac. 2023 Apr 17;36:100764. doi: 10.1016/j.lanwpc.2023.100764 (PMC10398586; doi:10.1016/j.lanwpc.2023.100764)
Supplement: IPD Protocol [file mmc2.pdf]

Research protocol

Comparing invasive pneumococcal disease and serotype 19A epidemiology in New Zealand and Australia

Research team

Nienke N. Hagedoorn
Department of Paediatrics, University of Otago, Christchurch, New Zealand

Andrew Anglemyer
- Health Intelligence Team, Institute of Environmental Science and Research, Wellington, New Zealand
- Department of Preventive and Social Medicine, University of Otago, Dunedin, New Zealand

Mica Hartley
Communicable Diseases and Surveillance Section, Australian Government Department of Health, Canberra, Australia

Tony Walls
Department of Paediatrics, University of Otago, Christchurch, New Zealand

Background

After introduction of the pneumococcal conjugate vaccine (PCV), invasive pneumococcal disease (IPD) and subsequent pneumococcal mortality has been reduced globally. Yet in 2015, pneumococcal infections are still estimated to cause 294,000 (range 192 –366,000) deaths in HIV-uninfected children under 5 years.(1) Some populations are at higher risk for IPD including young infants and specific ethnicities such as Aboriginal, Māori or Pacific people.(2, 3)

Several PCVs are commercially available and cover different serotypes (7-valent PCV (PCV7);10-valent PCV (PCV10) (Synflorix, GSK); 13-valent PCV (PCV13) (Prevenar 13, Pfizer)). In comparison with PCV10, PCV13 also includes the serotypes 3, 6A, and 19A. Previous studies comparing the impact of PCV10 and PCV13 programmes until 2017 have not found superiority for one of the two vaccines in the overall burden of pneumococcal disease.(4-6) The World Health Organization recommends that a switch in PCV programme should be made when epidemiology of IPD changes considerably.(6)

In the national immunisation programme of Australia PCV13 is used since 2011, whereas Aotearoa New Zealand (NZ) switched from PCV13 to PCV10 in 2017 (Appendix 1).(7, 8) When NZ switched to PCV10 in 2017 it was on the basis that immunogenicity studies suggested the inclusion of serotype 19F in PCV10 offers cross-protection for serotype 19A. Whether this cross-protection is sufficient to protect for infection by serotype 19A has been questioned.(9, 10) A recent NZ study showed that serotype 19A in IPD patients increased from 12% in 2017 to 21% in 2020 with overall IPD incidence remaining similar. Furthermore, half of the hospital admissions in children <5 years with IPD was due to serotype 19A, and penicillin-resistance in serotype 19A increased to 60%.(10) Recently, this trend of increasing serotype 19A infections has been noticed in several other countries around the world who use PCV10 on their infant immunisation schedule.(11-15)

Australia and New Zealand have had similar experiences in the COVID-19 pandemic with relatively low case numbers compared to other countries. As containment measures in the COVID-19 pandemic have shown to reduce IPD (16), Australia and New Zealand are relatively comparable in their IPD incidence. To our knowledge, no previous study has examined the influence of different

childhood immunisation programmes on IPD rate in Oceania. In this study, we aim to compare IPD incidence between Australia and New Zealand in 2017-2021, with a focus on young age, serotype 19A and ethnicity.

Study objective

To compare IPD incidence and epidemiology between Australia and New Zealand in 2017-2021, with a focus on ethnicity, age and serotype 19A.

Methods

Study design: This is an ecological study based on surveillance data from Australia and NZ

Data collection: We will collect IPD case notification data from national surveillance institutes (National Notifiable Diseases Surveillance System (NNDSS), EpiSurv) and merge those with census data that will be derived from Australian Bureau of Statistics and Statistics New Zealand. In NZ, all laboratories contribute to the surveillance system.

IPD is defined as a laboratory confirmed infections of *S. pneumoniae* if isolated from a normally sterile site or detected with nucleic acid testing from a sterile site. To be able to compare cases between Australia and NZ, cases detected from a newer generation *S. pneumoniae* antigen test on cerebrospinal fluid or pleural fluid will be excluded (NZ only).(17, 18)

National coverage of childhood immunisation data will be extracted from the National Immunisation registers in NZ and Australia. Fully immunised was defined as eligible children who have completed all of their age-appropriate immunisations by the time they turned the milestone age.

Sample size: This is a descriptive study so a power calculation is not required. Preliminary data show us that in 2019 overall IPD rates per 100,000 for Australia and New Zealand are on average 8.4 (95%CI 8.1-8.8), and 9.9 (95%CI 9.1-10.8), respectively. Therefore, this study will provide estimates for IPD rates with sufficient certainty.

Data analysis: First, we will compare overall IPD rates per 100,000 and the proportion of cases with serotype 19A between Australia and New Zealand between 2017-2021. Second, we will perform a stratified analysis for ethnicity: Indigenous (Aboriginal and Torres strait islander) versus non-Indigenous for Australia, and Māori and Pacific vs other for New Zealand. Next, we will analyse of IPD rates and serotype 19A rates in different age groups. Furthermore, we will explore whether differences in IPD rates can be explained by differences in national immunisation coverage for pneumococcal vaccinations. In a sensitivity analysis, we will repeat all the analyses only including non-Indigenous children in Australia because Indigenous children receive additional doses of PCV in certain regions (Appendix 1). Cases with missing indigenous status were counted as non-indigenous.

Ethical considerations

We will apply for ethical approval for this study from the University of Otago Human Ethics Committee. This study is a low-risk research project as it does not involve direct patient interactions and involves the use of anonymized data. The aggregated data from National Surveillance systems will not contain any identifiable variables.

Timeline

April 2022: literature review, ethics approval
May 2022: data analysis, data interpretation
June 2022: drafting manuscript, dissemination of results

Impact

Epidemiological and surveillance studies such as our project, are crucial to evaluate vaccine strategies and to inform public health organizations on vaccine policies to improve health outcomes. This research will provide insight in differences of IPD and serotype rates and the influence of the PCV10 and PCV13 programmes. This study will provide us with an opportunity to compare PCV10 and PCV13 programmes based on real-life data. In addition, we will evaluate the IPD rate in specific ethnic groups who suffer from health inequalities. In these groups, it is even more essential to evaluate the effect of current vaccine strategies. Also, this project will provide essential data on the future decision of vaccine programmes in New Zealand and Australia.

Dissemination

The results of this study will be disseminated in presentations at scientific meetings, international peer-reviewed journals, and the IPD surveillance working group in Australia and New Zealand.

Relevance to Māori health

Indigenous populations in New Zealand and Australia, along with Pacific peoples have a much higher burden of disease for IPD.(2, 3, 19) For instance, two-thirds of all IPD cases in 2020 among children aged <5 years were children of Māori (39%) or Pacific (28%) ethnicity.(10) Australia provides two additional PCV23 (Pneumovax 23) doses for Aboriginal and Torres Strait Islander children, from 4 years of age.(7) New Zealand's programme, however, does not have additional doses for Māori or Pacific people. In this study, we will evaluate the IPD rate in specific ethnic groups including Māori as it is even more essential to evaluate the effect of current vaccine strategies in groups at higher-risk for IPD. This study has the potential to guide future decisions regarding vaccine strategies for Māori.

Māori consultation will be requested following the procedure by the Otago university.

Appendix 1 – Important changes in immunisation schedules for children in Australia and New Zealand over time

Australia (7, 20)

2001-Jun	Introduction PCV7 in Indigenous children and children with high-risk medical conditions	Indigenous: PCV 7 at 2, 4 and 6 months (PCV7), in high-incidence jurisdictions* PPV23 at 18-24 months, Medically high-risk: PCV7 at 2, 4, 6 and 12 months, PPV23 at 5 years
2005-Jan	Universal 3 dose program PCV7	2, 4 and 6 months (PCV7), with catch-up doses in children <2 years
2011-Jul	PCV13 replaced PCV7	Catch-up dose of PCV13 for children 12-35 months. Indigenous: in high-incidence jurisdictions PCV13 replaced PPV23 at 18-24 months.
2018-Jul	PCV13 at different timings, change to 2+1	PCV13 at 2, 4 and 12 months. Indigenous children in high-incidence jurisdictions*, and children at medically higher-risk: PCV 13 at 2, 4, 6 and 12 months (3+1)

*High-incidence jurisdictions: Northern Territory (NT), South Australia (SA), Queensland (QLD) and Western Australia (WA).
Indigenous: includes Aboriginal and Torres Strait Islander

New Zealand (8)

2006	PCV7 for high-risk children	
2008-Jun	Universal PCV7	PCV7 at 6 weeks, 3, 5 months and 15 months
2011	PCV10 replaced PCV7	For high-risk children: PCV13 replaced PCV7
2014	PCV13 replaced PCV10	
2015	PCV13 available for patients of any age with certain high-risk conditions	
2017	PCV10 replaced PCV13	PCV13 continues for high-risk individuals
2020	PCV10 dosing change: 2-dose primary schedule plus booster dose given at 6 weeks, 5 months and 12 months (2+1)	For high-risk infants: PCV13 remained at 3-dose schedule plus booster for high-risk infants (6 weeks, 3, 5 and 12 months)

References

1. Wahl B, O'Brien KL, Greenbaum A, Majumder A, Liu L, Chu Y, et al. Burden of Streptococcus pneumoniae and Haemophilus influenzae type b disease in children in the era of conjugate vaccines: global, regional, and national estimates for 2000–15. The Lancet Global Health. 2018;6(7):e744-e57.
2. Meder KN, Jayasinghe S, Beard F, Dey A, Kirk M, Cook H, et al. Long-term Impact of Pneumococcal Conjugate Vaccines on Invasive Disease and Pneumonia Hospitalizations in Indigenous and Non-Indigenous Australians. Clinical Infectious Diseases. 2020;70(12):2607-15.
3. Petousis-Harris H, Howe AS, Paynter J, Turner N, Griffin J. Pneumococcal Conjugate Vaccines Turning the Tide on Inequity: A Retrospective Cohort Study of New Zealand Children Born 2006–2015. Clinical Infectious Diseases. 2019;68(5):818-26.
4. de Oliveira LH, Camacho LAB, Coutinho ESF, Martinez-Silveira MS, Carvalho AF, Ruiz-Matus C, et al. Impact and Effectiveness of 10 and 13-Valent Pneumococcal Conjugate Vaccines on Hospitalization and Mortality in Children Aged Less than 5 Years in Latin American Countries: A Systematic Review. PLOS ONE. 2016;11(12):e0166736.
5. Naucler P, Galanis I, Morfeldt E, Darenberg J, Örtqvist Å, Henriques-Normark B. Comparison of the Impact of Pneumococcal Conjugate Vaccine 10 or Pneumococcal Conjugate Vaccine 13 on Invasive Pneumococcal Disease in Equivalent Populations. Clin Infect Dis. 2017;65(11):1780-9.
6. World Health Organization. Pneumococcal conjugate vaccines in infants and children under 5 years of age: WHO position paper –February 2019 Weekly Epidemiological Record,. 2019;94(08):85-103.
7. Department of Health Australia. Vaccine history timeline [Available from: https://www.health.vic.gov.au/immunisation/vaccine-history-timeline.
8. New Zealand Ministry of Health. Immunisation Handbook 2020 [Available from: https://www.health.govt.nz/our-work/immunisation-handbook-2020/16-pneumococcal-disease.
9. Isturiz R, Sings HL, Hilton B, Arguedas A, Reinert R-R, Jodar L. Streptococcus pneumoniae serotype 19A: worldwide epidemiology. Expert Review of Vaccines. 2017;16(10):1007-27.
10. Anglemyer A, McNeill A, DuBray K, Sonder GJB, Walls T. Invasive Pneumococcal Disease: Concerning Trends in Serotype 19A Notifications in New Zealand. Clinical Infectious Diseases. 2021.
11. Desmet S, Verhaegen J, Van Ranst M, Peetermans W, Lagrou K. Switch in a childhood pneumococcal vaccination programme from PCV13 to PCV10: a defendable approach? The Lancet Infectious Diseases. 2018;18(8):830-1.
12. Desmet S, Lagrou K, Wyndham-Thomas C, Braeye T, Verhaegen J, Maes P, et al. Dynamic changes in paediatric invasive pneumococcal disease after sequential switches of conjugate vaccine in Belgium: a national retrospective observational study. Lancet Infect Dis. 2021;21(1):127-36.
13. Brandileone MC, Almeida SCG, Minamisava R, Andrade AL. Distribution of invasive Streptococcus pneumoniae serotypes before and 5 years after the introduction of 10-valent pneumococcal conjugate vaccine in Brazil. Vaccine. 2018;36(19):2559-66.
14. Brandileone MC, Almeida SCG, Bokermann S, Minamisava R, Berezin EN, Harrison LH, et al. Dynamics of antimicrobial resistance of Streptococcus pneumoniae following PCV10 introduction in Brazil: Nationwide surveillance from 2007 to 2019. Vaccine. 2021;39(23):3207-15.
15. Severiche-Bueno DF, Severiche-Bueno DF, Bastidas A, Caceres EL, Silva E, Lozada J, et al. Burden of invasive pneumococcal disease (IPD) over a 10-year period in Bogotá, Colombia. Int J Infect Dis. 2021;105:32-9.
16. Brueggemann AB, Jansen van Rensburg MJ, Shaw D, McCarthy ND, Jolley KA, Maiden MCJ, et al. Changes in the incidence of invasive disease due to Streptococcus pneumoniae, Haemophilus influenzae, and Neisseria meningitidis during the COVID-19 pandemic in 26 countries and territories in the Invasive Respiratory Infection Surveillance Initiative: a prospective analysis of surveillance data. Lancet Digit Health. 2021;3(6):e360-e70.
17. Department of Health Australia. Pneumococcal disease (invasive) case definition [updated 12/03/2004. Available from:

https://www1.health.gov.au/internet/main/publishing.nsf/Content/cda-surveil-nndss-casedefs-cd_pnuemo.htm.
18. Ministry of Health. Communicable Disease Control Manual - Invasive pneumococcal disease [updated 13/05/201922/03/2022]. Available from: https://www.health.govt.nz/our-work/diseases-and-conditions/communicable-disease-control-manual/invasive-pneumococcal-disease.
19. Baker MG, Barnard LT, Kvalsvig A, Verrall A, Zhang J, Keall M, et al. Increasing incidence of serious infectious diseases and inequalities in New Zealand: a national epidemiological study. Lancet (London, England). 2012;379(9821):1112-9.
20. National Centre for immunisation research and surveillance. Significant events in pneumococcal vaccination practice in Australia [updated July 2020. Available from: https://ncirs.org.au/sites/default/files/2020-07/Pneumococcal-history-July-2020.pdf.
